# Basal Ganglia Ischemic Stroke: The Unforeseen Progression of Tuberculosis Epididymo-Orchitis

**DOI:** 10.7759/cureus.46640

**Published:** 2023-10-07

**Authors:** Abraham Mengstu, Seti Belay, Mathew N Chakko, Adithya Bala

**Affiliations:** 1 Radiology, Ascension Providence Hospital/Michigan State University College of Human Medicine, Southfield, USA; 2 Radiology, Rochester Regional Health/Rochester General Hospital, Rochester, USA

**Keywords:** central nervous system infections (cns), intracranial tuberculoma, tuberculous meningitis (tbm), complicated meningitis, mycobacterium tuberculous, ischemic cerebrovascular disease, basal ganglia infarction, anti-tuberculosis therapy, neuroradiology, global health

## Abstract

Tuberculosis is an infectious disease with broad pulmonary and extrapulmonary clinical manifestations. Central nervous system tuberculosis (CNS-TB) is a complex extrapulmonary infection known for its diverse clinical features including meningitis, tuberculoma, and spinal arachnoiditis. Particularly, tuberculosis meningitis can further lead to complications such as ischemic stroke.

This article presents a challenging case of a 35-year-old male patient initially diagnosed with epididymo-orchitis, followed by viral-like central nervous system symptoms, ultimately complicated by tuberculosis meningitis and basal ganglia ischemic stroke.

This case presentation underscores the diagnostic complexities associated with CNS-TB and emphasizes on the critical need for heightened awareness of the wide-ranging clinical presentations that can potentially delay early disease recognition and management.

## Introduction

Tuberculosis (TB) is a pressing global health challenge with profound implications for public health, society, and economies [1]. The widespread occurrence of the disease, together with the emergence of diagnostic techniques like the nucleic acid amplification tests introduced in 2009, emphasizes TB's persistent importance and the relentless pursuit to understand and manage it. Despite advancements in medical science, TB's widespread impact necessitates ongoing vigilance, research, and public health interventions to curtail its global ramifications [1].

TB remains a significant public health burden with an estimated death of about 1.6 million in 2022 despite concerted global anti-tuberculosis measures [2]. In the United States, for example, the introduction of effective anti-tuberculosis medications and the implementation of public health measures, such as contact tracing and targeted testing, led to a decline in TB cases starting in the mid-20th century to about 2.7, 2.4 and 2.5 cases per 100,000 in 2019, 2021 and 2022 respectively [1], with a slight increase in 2022 attributed to possible coronavirus disease 2019 (COVID-19) pandemic-related obstacles in implementing anti-tuberculosis public health measures. 

Although tuberculosis is often thought of as an infectious disease with progressive pulmonary involvement (pulmonary tuberculosis), it can possibly affect any organ system (extrapulmonary tuberculosis). However, the prevalence of these clinical presentations varies by geography. For example, this is illustrated in a six-year retrospective study conducted by Rolo et al. in a Spanish tertiary care facility published in 2023 [3]. Among 398 confirmed cases of tuberculosis in this study, 20.9% (n = 83) were diagnosed with extrapulmonary tuberculosis, 60.8% (n = 242) with pulmonary tuberculosis, 8.5% (n = 34) with disseminated tuberculosis, and 3.8% (n = 15) with concurrent extrapulmonary-pulmonary tuberculosis with no reported cases of central nervous system (CNS) TB infection [3].

Mycobacterium tuberculosis, a bacterium with epidemiologic preponderance in low- and middle-income countries [4], spreads through aerosolization of droplets leading to respiratory infection [5]. In immunocompetent individuals, the mycobacterium is typically encapsulated within granulomas, known as Ghon’s foci/lesions by the respiratory macrophage system. The broader term Ghon's complex encompasses the initial focus alongside ipsilateral hilar lymphadenopathy. These encapsulated granulomas may either heal with residual calcification, remain dormant and house latent Mycobacterium tuberculosis poised for activation when the host is in an immunosuppressed state, or progress into active diseases like cavitation. Conversely, individuals with compromised immune systems are more prone to disseminated tuberculosis upon initial entry of the bacterium. 

Following entry into the host, the bacterium can spread via a hematogenous route to distant sites, rendering every organ, including the CNS, vulnerable to potential infection. 

In the following sections, we outline an unusual tuberculosis case, followed by a discussion of the diagnostic challenges, imaging findings, and their impact on patient care.

## Case presentation

**Figure 1:**
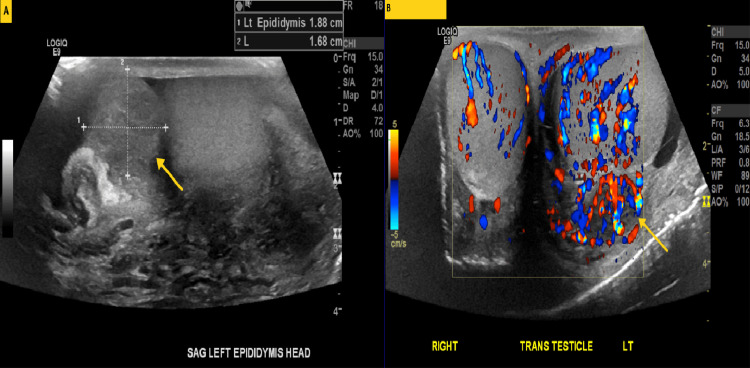
Representative grayscale (A) and color doppler (B) sonographic images through the left epididymis/testicle demonstrate enlarged and hypervascular left testicle and epididymis suggestive of epididymo-orchitis.

A 35-year-old male without significant past medical history was presented to emergency with testicular pain and swelling. A high riding and tender left testicle was appreciated on physical examination. Ultrasound evaluation demonstrates enlargement, thickening and irregularity of the left testicle and epididymis with associated increased vascular flow to the ipsilateral testicle and epididymis suggestive of epididymo-orchitis (Figure 1). The patient was diagnosed with acute epididymo-orchitis and discharged home with oral antibiotics. His scrotal swelling was waxing and waning and led to trial of multiple antibiotics.

Seven months after the first presentation, the patient presented to the hospital with complaints of headache, neck stiffness and low-grade fever for 36 hours. Physical examination was unremarkable. With the impression of meningitis, the patient was empirically initiated on an intravenous regimen comprising dexamethasone, vancomycin, ceftriaxone, and acyclovir.

Subsequently, diagnostic lumbar puncture was performed and cerebrospinal fluid (CSF) was sent for analysis revealing elevated protein level of 73.9 mg/dL, low glucose level of 22 mg/dL, and elevated white blood cell count of 44 cells/dL (34% neutrophils and 66% mononuclear cells). Cerebrospinal fluid was negative for gram and acid-fast staining. Preliminary culture results for fungi, acid fast bacilli and other bacteria as well as polymerase chain reactions (PCR) for herpes simplex virus type 1, herpes simplex virus type 2 and West Nile viruses were nonrevealing. Serology tests for syphilis, HIV 1, and HIV 2 were also negative. With CSF findings suggestive of acute lymphocytic meningitis and improvement in patient symptoms, the patient was discharged after a three-day course of intravenous acyclovir and a four-day course of intravenous steroid and antibiotics, without further need for antibiotics, antivirals, or steroids. No neuroimaging was obtained during this emergency presentation or during his inpatient stay. 

The following day, the patient returned to the emergency department with persistent symptoms of headache, neck stiffness, and a low-grade fever. The patient was again discharged from emergency with methylprednisolone tablets for his pain and low-grade fever. However, he was brought back to the emergency the next day due to confusion. At this visit, Code stroke was activated and neuroimaging was perused with non-contrast-enhanced CT scan of the head/brain and CT angiogram of the head/neck. 

**Figure 2:**
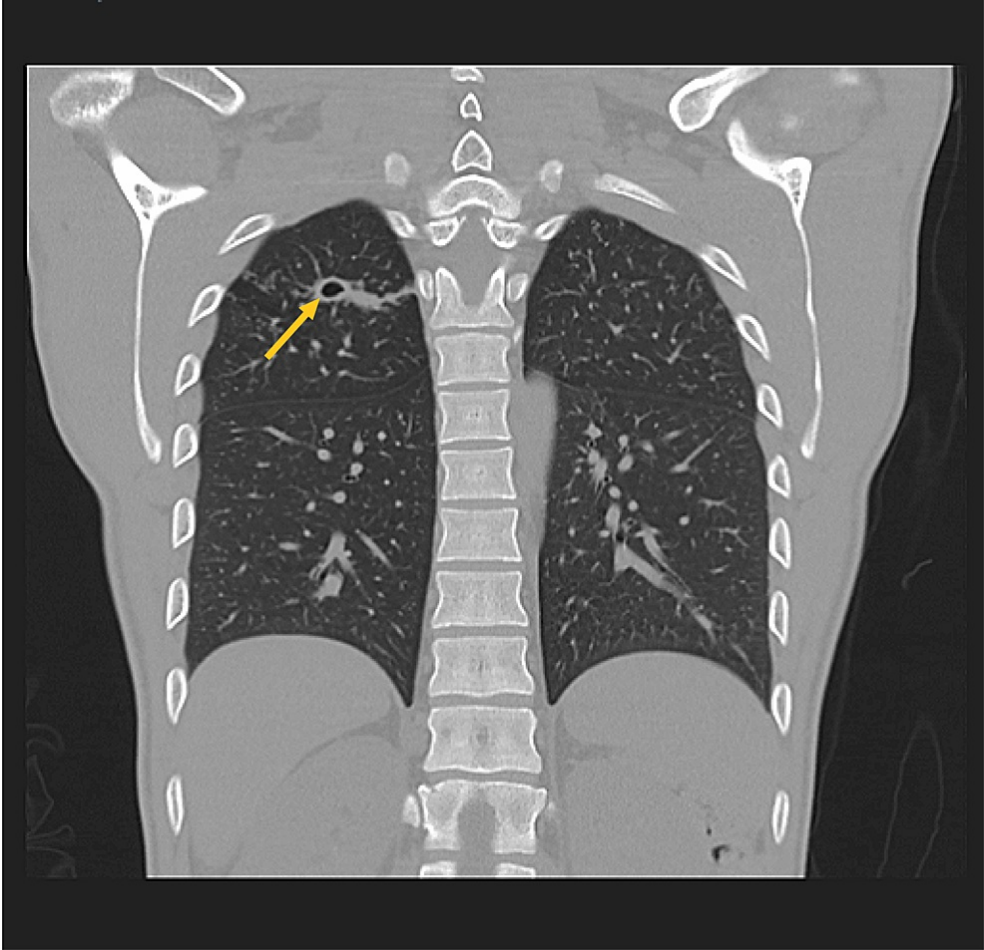
Representative coronal computed tomographic (CT) image of the chest demonstrates right upper lobe cavitary lesion compatible with infectious causes including mycobacterial and fungal etiologies.

Non-contrast-enhanced head/brain CT and head/neck CT angiogram revealed mild enlargement of the lateral ventricles, along with increased periventricular hypoattenuation that was unusual for the patient's age raising the concern of possible meningitis (image not provided). Incidentally, a small cavitary lesion was identified in the right upper lobe on head/neck CT angiogram. This led to dedicated non-contrast enhanced thoracic CT imaging with similar findings, suggesting the diagnosis of tuberculosis infection (Figure 2).

Disseminated tuberculosis was considered and the patient was admitted to the intensive care unit (ICU). Patient was initiated on the anti-tuberculosis regimen of rifampin, isoniazid, pyrazinamide, and ethambutol (RIPE) therapy along with intravenous dexamethasone and empiric intravenous antibiotics. Bronchoalveolar lavage was performed and acid-fast bacilli (AFB) were seen on microscopy. Repeat cerebrospinal fluid analysis revealed elevated protein levels at 81.8 mg/dL, low glucose levels at 14 mg/dL, and an increased white blood cell count at 76 cells/dL (19% neutrophils and 77% lymphocytes). Cerebrospinal tests were negative for enterovirus, herpes simplex virus type 1, herpes simplex virus type 2, Toxoplasma gondii, varicella-zoster virus, Cryptosporidium, cytomegalovirus, Histoplasma capsulatum, and West Nile virus.

**Figure 3:**
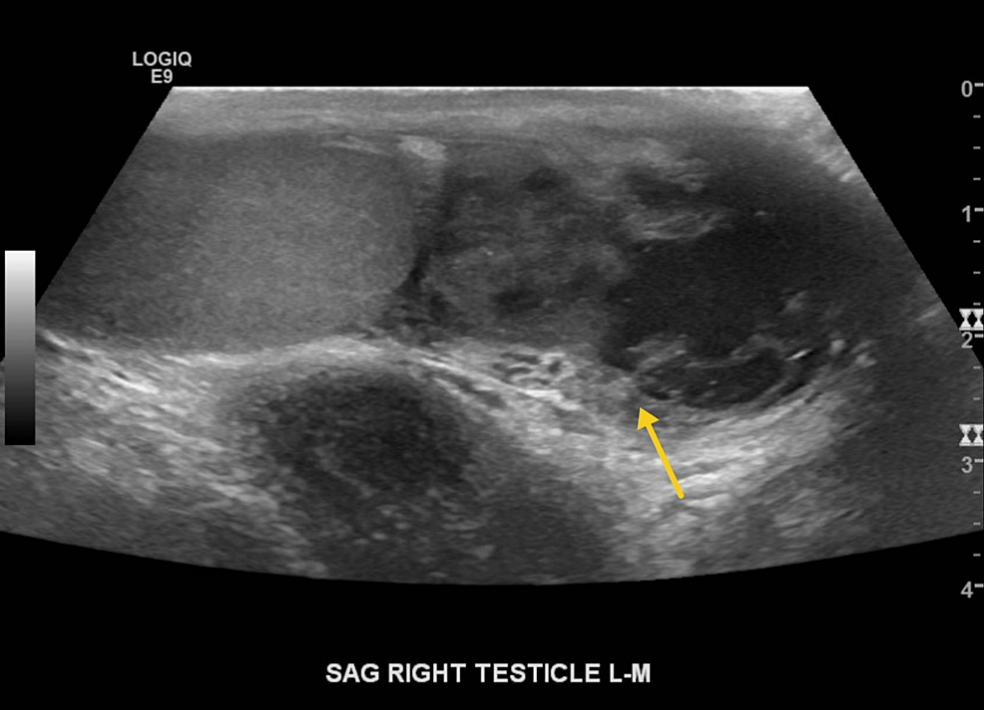
A representative grayscale sonographic image through the right testicle demonstrates development of new heterogeneous enlargement of the right epididymis with an extratesticular complex masslike area on the right testicle/epididymis.

Repeated sonographic imaging of the scrotum was performed demonstrating heterogeneous enlargement of the bilateral epididymides with a complex extra-testicular mass-like area noted on the right testicle/epididymis (Figure 3). The right scrotal testicular/epididymis findings were new for the previous sonographic evaluation. Subsequently, the left scrotum was explored and a biopsy of the left epididymal mass was obtained demonstrating AFB and nucleic acid amplification test (NAAT) positive for tuberculosis. 

Once microbiology evidence was obtained, antibiotics were discontinued and the patient was continued on anti-tuberculosis medications. While on inpatient anti-tuberculosis medications, the patient's symptoms improved and his headache gradually diminished. He was discharged on RIPE therapy under health department surveillance. Patient remained adherent to RIPE therapy as an outpatient.

About 10 days from discharge, family members brought the patient back for progressively worsening confusion and forgetfulness, headaches, and neck discomfort. Physical examination was unremarkable. 

**Figure 4:**
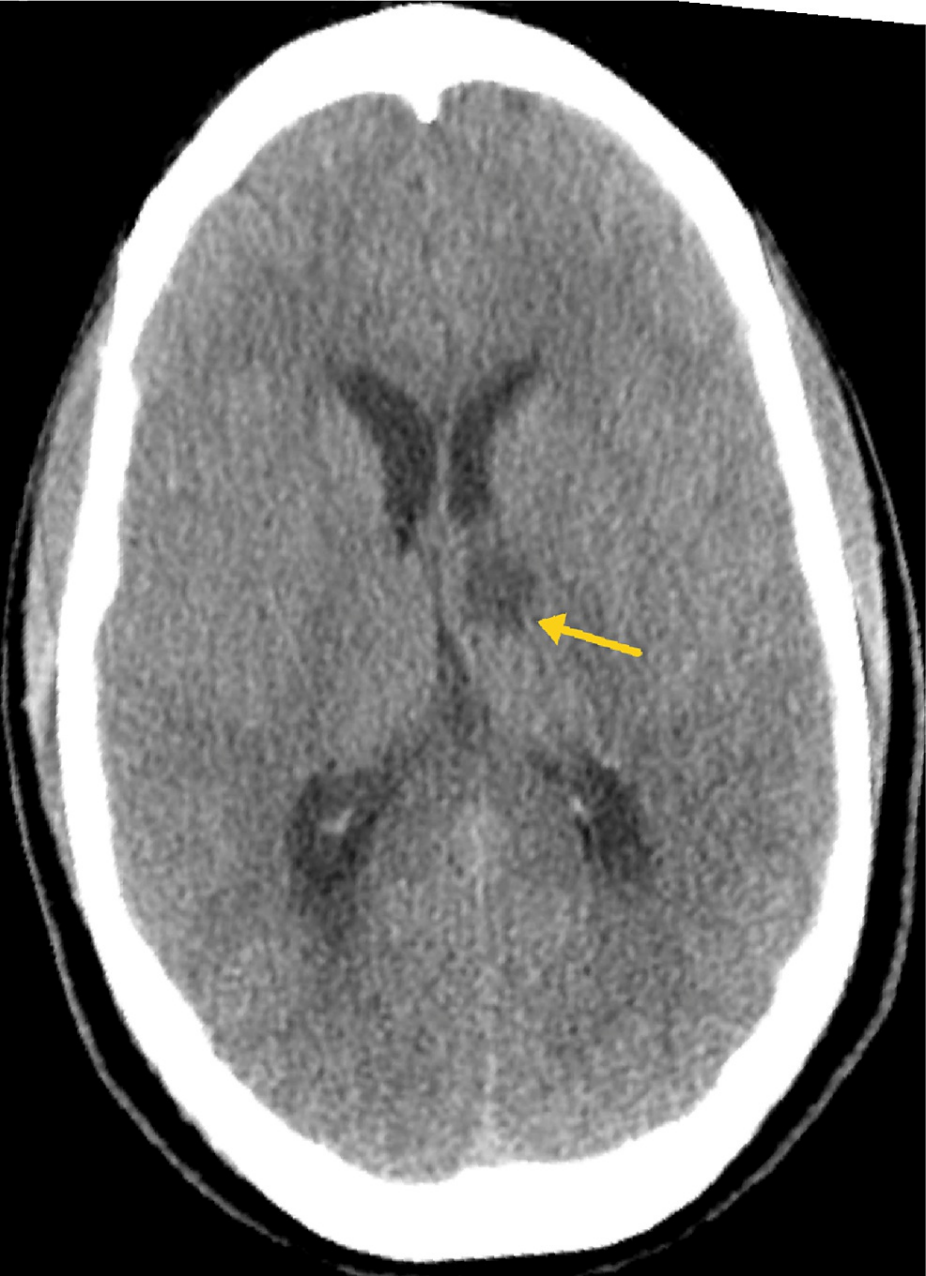
Representative axial computed tomographic (CT) image through the level of the basal ganglia demonstrates new hypodensity in the anterior left thalamus concerning for ischemic infarct.

**Figure 5:**
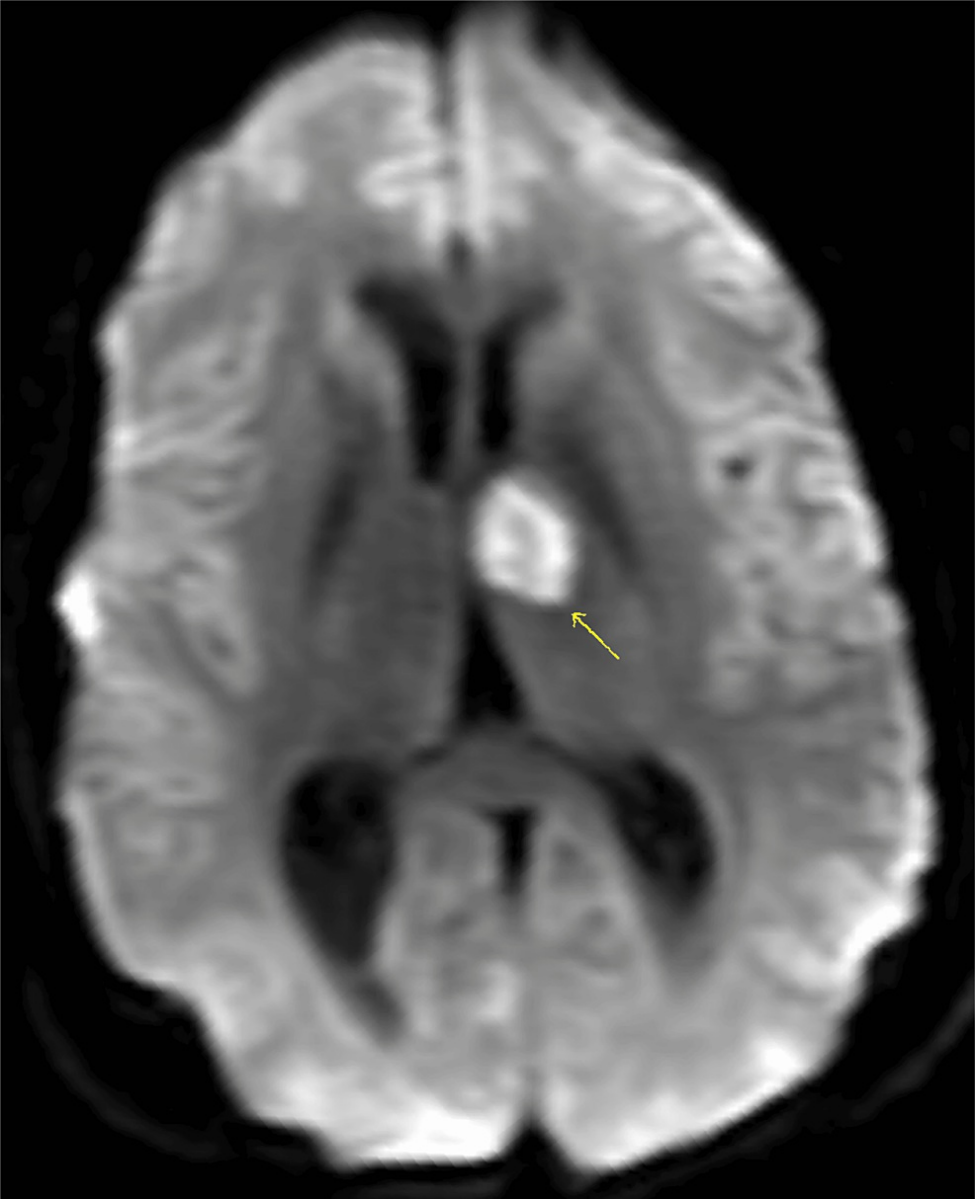
Representative diffusion-weighted (DW) image through the level of the basal ganglia demonstrates restricted diffusion in the anterior left thalamus compatible with acute/early subacute infarct.

**Figure 6:**
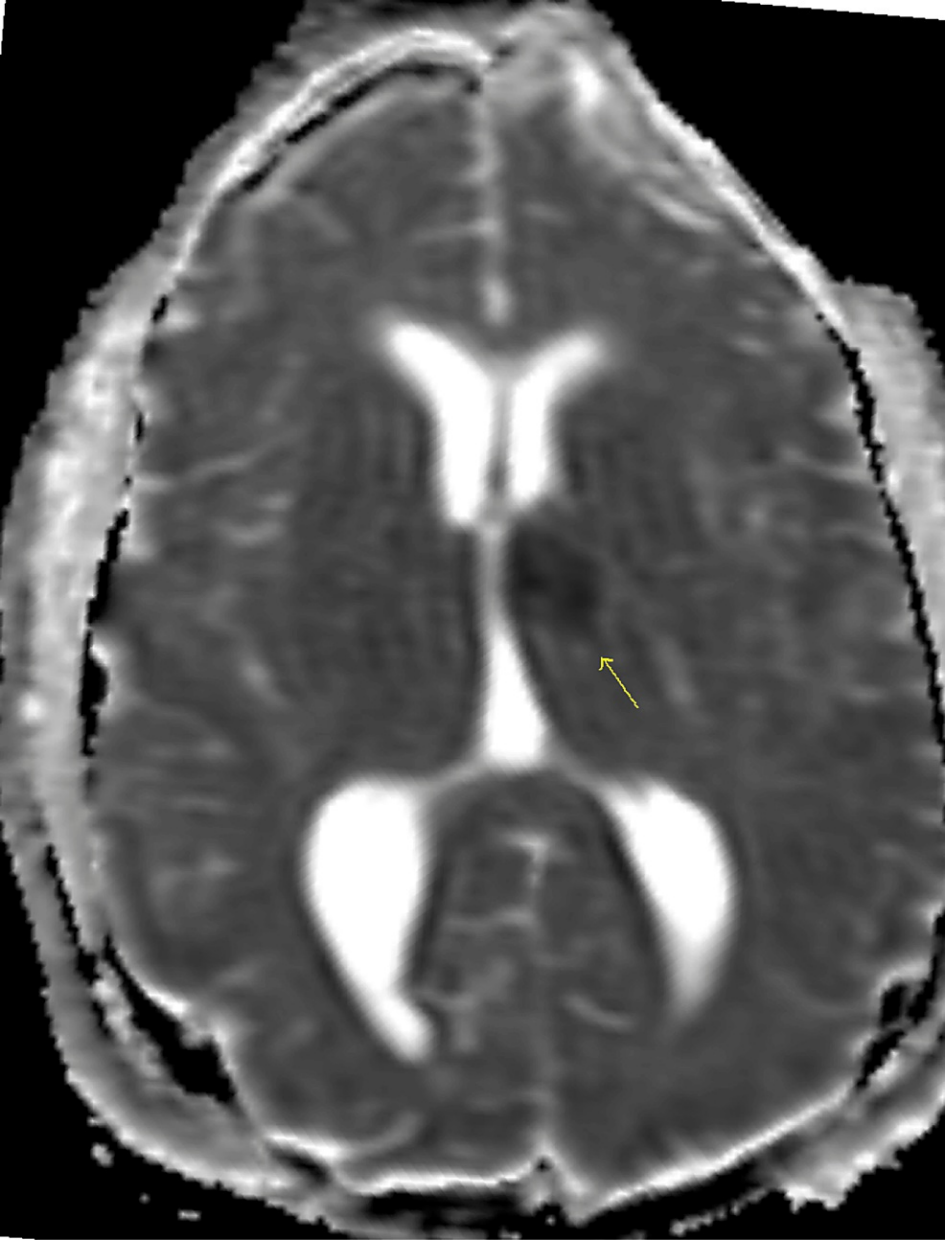
Representative apparent diffusion coefficient (ADC) map through the level of the basal ganglia demonstrates region of low ADC value in the anterior left thalamus compatible with acute/early subacute infarct.

**Figure 7:**
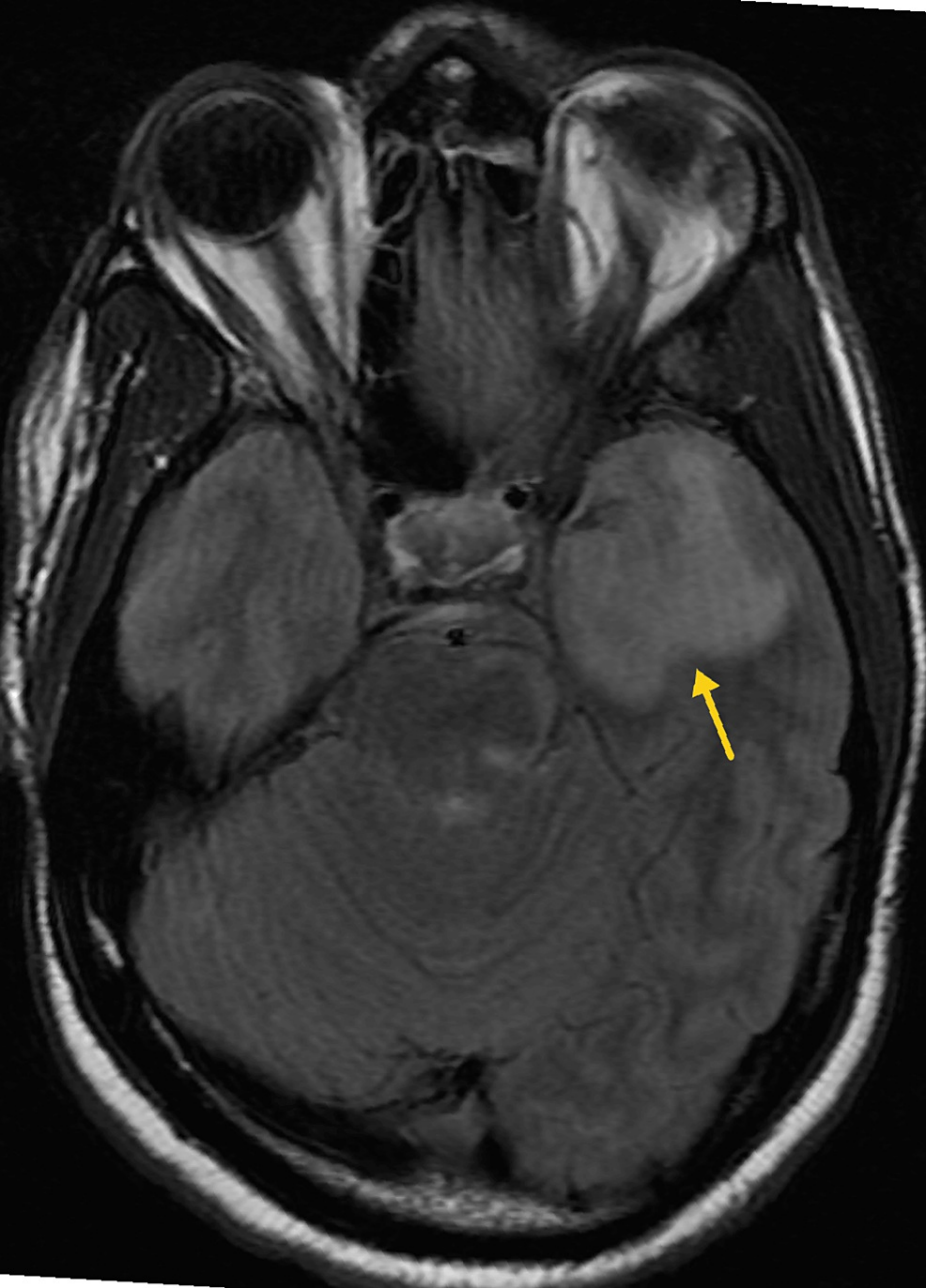
Representative axial fluid-attenuated inversion recovery (FLAIR) image through the level of the temporal lobes demonstrates extensive anterior left temporal edema consistent with cerebritis. Mild edema is also noted in the right temporal lobe.

**Figure 8:**
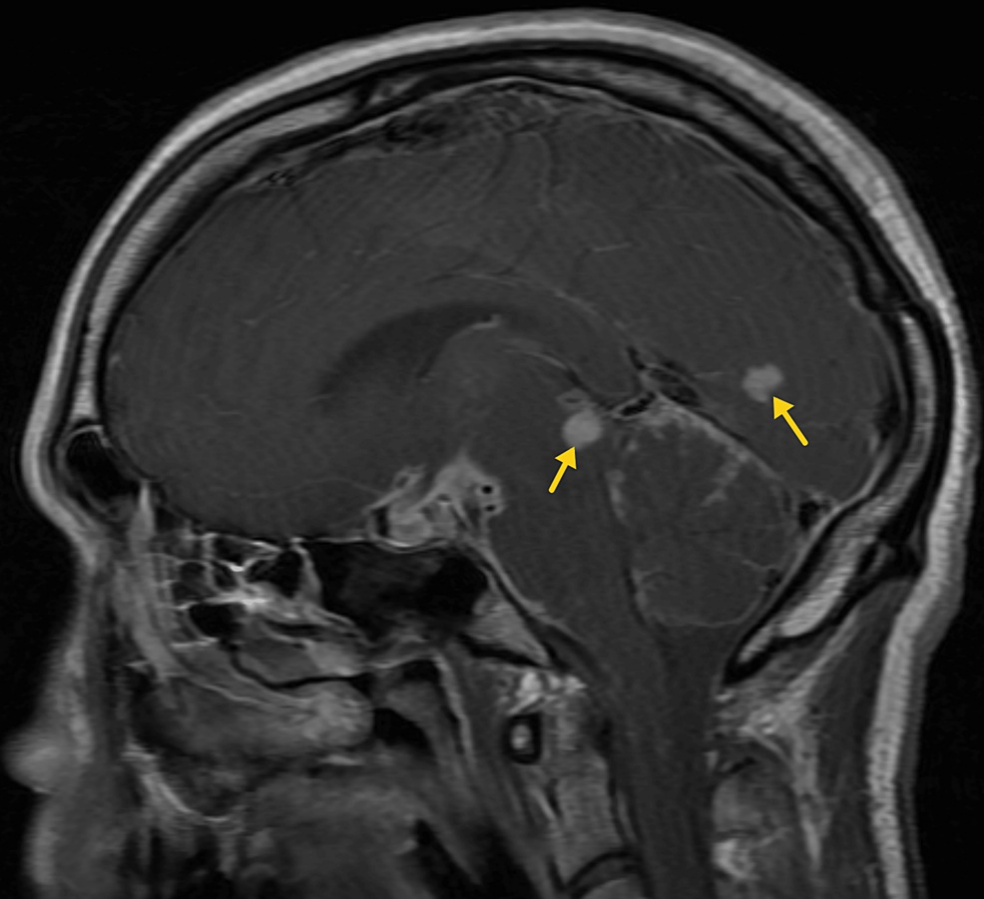
Representative post-gadolinium sagittal magnetic resonance imaging (MRI) demonstrates scattered supratentorial and infratentorial nodular intraparenchymal enhancements consistent with tuberculomas.

**Figure 9:**
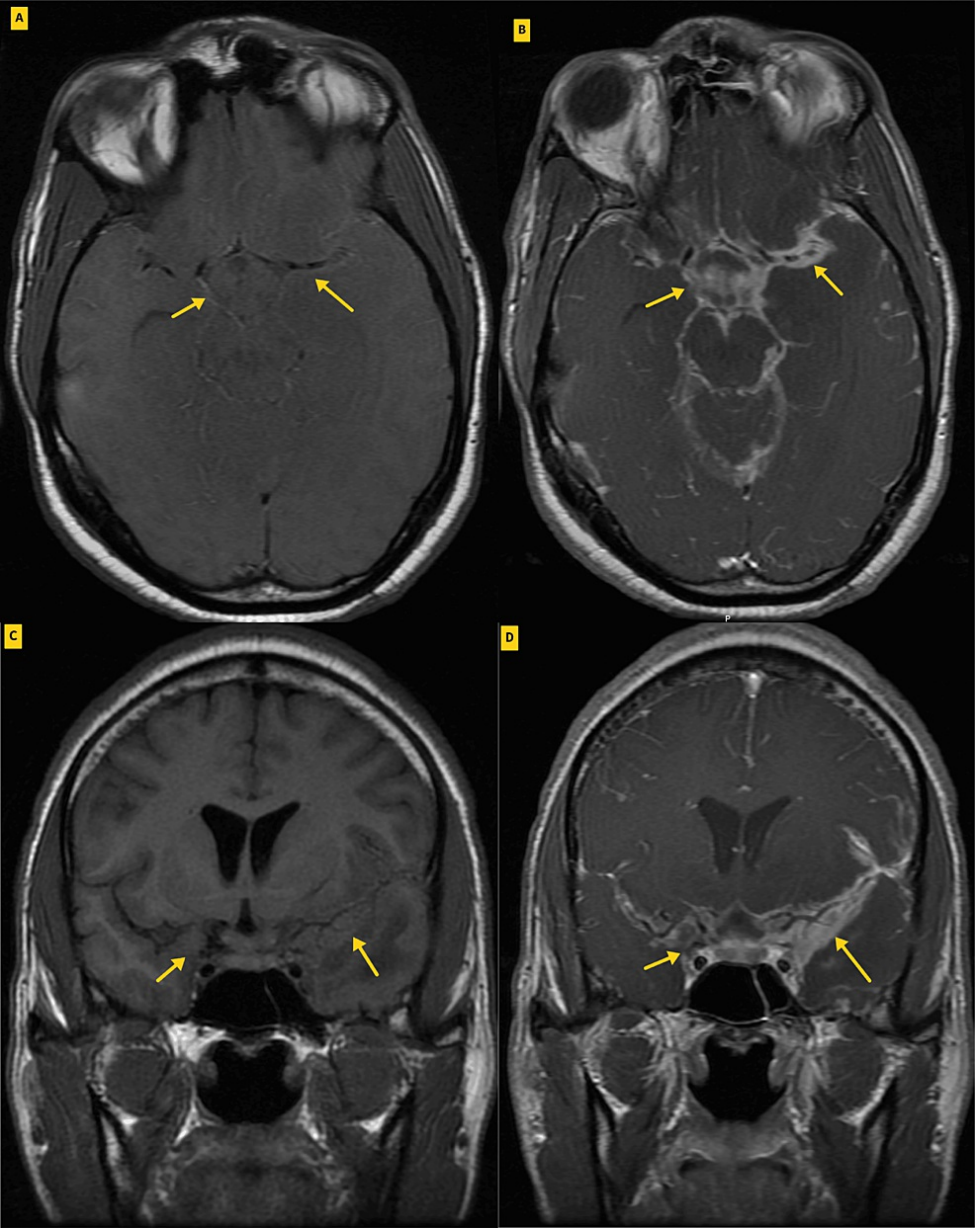
Representative axial (A and B) and coronal (C and D) pre-contrast (A and C) and post-gadolinium (B and D) magnetic resonance imaging (MRI) through the suprasellar cistern demonstrate diffuse leptomeningeal enhancement consistent with meningitis.

Subsequently obtained CT head/brain (limited images provided) revealed new development of hypodensity in the left thalamus (Figure 4) that was concerning for ischemic infarct. Stroke protocol and post-gadolinium MRI demonstrated a well-defined area of restricted diffusion in the anterior left thalamus (consistent with acute/early subacute infarct) (Figures 5, 6), extensive anterior left temporal and supraorbital frontal edema (consistent with cerebritis) (Figure 7), scattered nodular intraparenchymal enhancements throughout the supratentorial and infratentorial brain (consistent with tuberculomas) (Figure 8), and profound diffuse leptomeningeal enhancement thickest in the left sylvian fissure and basal cisterns with involvement of the bilateral internal auditory canals (consistent with meningitis) (Figure 9) with overall finding attributable to complicated tuberculosis meningitis.

While the patient was admitted, patient received multidisciplinary care with subsequently obtained neuroimaging findings unchanged. Patient’s mental status improved slowly and the patient was discharged with improvement.

## Discussion

CNS-TB is seen in 5% and 15% of tuberculosis cases among immunocompetent and immunocompromised individuals respectively [6]. Although concurrent presentation is a usual feature, CNS-TB is categorized primarily into either tuberculoma, spinal arachnoiditis or meningitis based on clinical features and imaging findings [6]. Central nervous system tuberculomas are granulomatous lesions of variable sizes and number with predilection for the frontal and parietal lobe brain parenchyma [6]. Patients with tuberculoma can present with nonspecific symptoms such as headaches or focal neurologic manifestations [6]. Intracranial and spinal arachnoid spaces can be involved with Mycobacterium tuberculosis, however, spinal arachnoiditis usually refers to tuberculosis infection of the spinal arachnoid space resulting in meningeal nodularity and matting of primarily lumbar nerve roots [6]. Tuberculous meningitis (TBM) is a severe manifestation of CNS tuberculosis accounting for a significant burden of morbidity and mortality [6,7]. It presents with prodromal symptoms and subacute to chronic headaches. Neck stiffness and positive Brudnizki’s signs are variable physical examination findings.

Significant neurological complications such as ischemic stroke, hydrocephalus, cerebritis, brain abscess, and empyema can be seen with complicated TBM [8-10]. These complications show regional variation in frequency. For example, a systematic review of studies with a total of 8460 tuberculosis adult and pediatric patients (age range two months to 85 years, 62.5% men) revealed an averaged point estimate of 0.3 tuberculosis-related stroke cases (95% CI, 0.26-0.33), ranging from 0.08 in Saudi Arabia to 0.56 in France, with overall trends for higher proportions in low- and middle-income countries [9]. 

While the detailed complex pathophysiology of TBM-associated ischemic stroke is beyond the scope of this case presentation, a complex interplay between the host's immune response and Mycobacterium tuberculosis have been implicated in the pathogenesis of TBM-associated ischemic stroke. Multiple pathways ultimately lead to the development of a combination of vasospasm, compression of arteries by the gelatinous inflammatory exudate, granulomatous vasculitis from direct mycobacterial invasion, and hypercoagulable state [9,11-13].

Hsieh et al. mapped areas of the brain commonly affected by TBM-associated stroke to the region of anterior basal ganglia [14]. Among 14 TBM-associated stroke cases, 75% of the infarctions were seen in the medial lenticulostriate and thalamo-perforating territories that were termed the “TB zone” compared to the “ischemic zone” in the regions of the lateral lenticulostriate, anterior choroidal and thalamogeniculate arteries [14]. However, subsequent studies attempting to replicate these distribution patterns failed to make similar conclusions. According to Tai et al. (51 TBM, 34 cases of TBM-associated infarctions), there was concurrent involvement of the “ischemic zone” and “ TB zone” in 20% of the cases, with only 6% of the cases confined to the “TB zone” [13]. Sy et al. extensively analyzed pooled data of a larger population size and mapped proportions of TBM-associated strokes to the respective brain areas and arterial territories (Figure 1) [9]. The highest proportions were seen with basal ganglia infarcts, followed by multifocal infarct and cortical/lobar involvement (0.6, 0.49 and 0.26 respectively) [9]. Lateral lenticulostriate, middle cerebral and medial lenticulostriate arteries were involved with 0.55, 0.37 and 0.31 respective proportions [9]. In their conclusion, Tai et al. suggested the need for describing TBM-associated strokes in terms of arterial involvement over the traditional way of localizing strokes into dichotomous TB or ischemic zones [13]. 

Distinguishing between TBM-associated ischemic stroke and other causes of cerebrovascular ischemic strokes, as well as other complications of TBM, is challenging due to the broad array of overlapping clinical features. For example, fever, headache, neck stiffness and altered sensorium were reported with 0.86, 0.76, 0.74 and 0.69 rates among 302, 158, 71 and 189 TBM-associated stroke patients respectively [9]. Focal neurologic symptoms such as hemiplegia/focal weakness, cranial nerve palsy and seizure were reported at 0.62, 0.42, and 0.26 rates among 288, 107 and 290 TBM-associated stroke cases respectively [9]; mortality and poor outcome rates of 0.22 and 0.51 were reported among a total of 473 and 284 TBM-associated stroke patients respectively [9]. In this particular patient, headaches and confusion were observed at different points in time.

Cerebrospinal fluid analysis showing lymphocytic pleocytosis also lacks specificity, as a broad range of fungal and viral infections can lead to a similar laboratory profile. Acid-fast bacilli staining, while a rapid and cost-effective test, demonstrates poor diagnostic performance (with sensitivity ranging from 45% to 80% and positive predictive values from 50% to 80%) according to the Centers for Disease Control and Prevention [15]. The NAAT offers the advantage of delivering results a week before culture, with a sensitivity rate of 80% to 90% [15]. However, its utility is limited in regions where nontuberculous mycobacterial infections are prevalent. Despite these advances, culture remains the established benchmark for diagnosing TBM or other mycobacterial infections.

Neuroimaging plays a vital role in suspected CNS-TBM and/or its complications [6]. Contrast-enhanced computed tomography and magnetic resonance imaging (MRI) display varying degrees of leptomeningeal enhancement, primarily concentrated in the basal cistern. However, these imaging observations are not specifically indicative of Mycobacterium tuberculosis. Conditions like central nervous system toxoplasmosis, cryptococcosis, primary or secondary lymphomas, pyogenic meningitis, and nontuberculous mycobacterial infections (such as Mycobacterium bovis) can exhibit similar imaging characteristics. Additional imaging findings such as brain abscess, subdural empyema, infarct, and tuberculoma can also be seen in complicated meningitis [6].

In this specific case, the patient exhibited significant leptomeningeal enhancement, strikingly in the basal cistern and left sylvian fissure (Figure 9). Tuberculomas were also evident in both the supratentorial and infratentorial brain parenchyma (Figure 8). Furthermore, cerebritis involving predominantly the left frontal and temporal lobes was observed (Figure 7). Additionally, an acute/subacute ischemic stroke was noted in the left thalamic region (Figures 4, 5, 6). According to Hsieh et al. mapping, the anterior left thalamic ischemic stroke seen in this particular patient corresponds to the intersection area in the “TB zone” and “ischemia zone” with likely medial lenticulostriate and/or thalamo-perforating arteries involved [14].

Efforts were made to characterize sociodemographic, clinical, and imaging characteristics that predispose young individuals with tuberculosis meningitis to develop ischemic stroke. It was observed that patient age, elevated CSF white blood cell (WBC) count, and basal meningeal enhancement were independent predictors of TBM-associated ischemic stroke [16]. In our specific case, we observed concordant findings of an elevated CSF WBC count and basal meningeal enhancement. However, it is noteworthy that our patient was also presented with tuberculomas that were not found to be associated with TBM ischemic stroke [16]. 

While the incidence of TB in the United States has declined over the years, certain populations remain vulnerable, and the emergence of drug-resistant strains poses new challenges [1]. Foreign-born individuals, racial and ethnic minorities, individuals experiencing homelessness, and those with compromised immune systems face a higher risk of TB infection and disease. Furthermore, socioeconomic factors, such as poverty and limited access to healthcare, contribute to the persistence of TB in certain communities [1,17,18]. In our case, efforts to identify risk factors were inconclusive. The patient tested negative for HIV and had no history of homelessness or apparent contact with known TB patients. Consequently, it remains imperative for medical professionals to always remain vigilant.

Effectively managing ischemic stroke in TBM requires a collaborative, multidisciplinary effort involving neurologists, infectious disease specialists, and critical care teams. This approach aims to optimize anti-TB treatment, control inflammation, and manage vascular risk factors, all essential components in stroke management within TBM cases. 

## Conclusions

In summary, diagnosing CNS-TB remains challenging due to its diverse clinical presentation, nonspecific laboratory profiles, and inconclusive imaging findings. The presence of complications, such as ischemic stroke, further complicates the clinical picture. These diagnostic complexities are accentuated by declining incidence rates in certain regions, exacerbating the difficulties for healthcare professionals in achieving early diagnosis. Consequently, delays in the recognition and management of CNS-TB are frequent, resulting in heightened risks of mortality and morbidity. In this article, we highlighted a complex case involving the delayed diagnosis of tuberculosis epididymo-orchitis, which subsequently progressed to incidental pulmonary imaging findings. Notably, even after the identification of pulmonary and genitourinary tuberculosis, the diagnosis of CNS-TB was further delayed until the patient exhibited symptoms of basal ganglia stroke, despite frequent emergency visits prompted by nonspecific symptoms.

Therefore, heightened awareness among healthcare professionals is crucial in minimizing delays in diagnosis and management, reducing associated morbidity and mortality rates. Additionally, it plays a crucial role in preventing disease transmission within the public health domain. Hence, healthcare professionals must remain vigilant to effectively tackle the potential diagnostic challenges posed by CNS-TB.
